# Random Walks with Invariant Loop Probabilities: Stereographic Random Walks

**DOI:** 10.3390/e23060729

**Published:** 2021-06-08

**Authors:** Miquel Montero

**Affiliations:** 1 Departament de Física de la Matèria Condensada, Universitat de Barcelona (UB), Martí i Franquès 1, E-08028 Barcelona, Spain; miquel.montero@ub.edu; 2 Universitat de Barcelona Institute of Complex Systems (UBICS), Martí i Franquès 1, E-08028 Barcelona, Spain

**Keywords:** random walk, heterogeneous medium, survival analysis, hyperbolic geometry, elliptic geometry

## Abstract

Random walks with invariant loop probabilities comprise a wide family of Markov processes with site-dependent, one-step transition probabilities. The whole family, which includes the simple random walk, emerges from geometric considerations related to the stereographic projection of an underlying geometry into a line. After a general introduction, we focus our attention on the elliptic case: random walks on a circle with built-in reflexing boundaries.

## 1. Introduction

The random walk (RW) is one of the most widely used mathematical models to express the irregular evolution of certain physical systems [1,2,3]. In its simplest form, a RW can be viewed as a succession of either left-ward or right-ward jumps in the position of a particle according to certain probabilities. These one-step transition probabilities need not be constant to grant that the stochastic process thus defined belongs to the class of Markov chains [4]: the probability should depend only on properties linked to the current status of the walker, as the calendar time if they suffer from aging [5], or the geographic location when particles propagate through an inhomogeneous medium [6,7].

A recurrent consequence of time- and site-dependent transition probabilities is the appearance of some directionality in the evolution of the system: sometimes in the form of an exogenous bias, sometimes in the form of a restoring force. The inhomogeneous transition probabilities that we are going to consider here are affected by this peculiarity but not in a purely local way: we demand that the probability of performing a closed loop be a function of the number of steps taken exclusively. This means that a fluctuation that reverts the particle to the stating point is equally as likely as the mirror-reversed one, irrespective of the initial location chosen.

Despite this, except in the case of the simple RW, the closest-neighbor transition probabilities depend on the explicit locations of both (starting and ending) sites, indicating the presence of some geometry beyond the topological structure of the state space of the system. In a previous work [8], we considered, in detail, the case in which the underlying metric space was hyperbolic: on the one side, there was evidence pointing to the presence of hyperbolic geometries in technological, biological, or social complex networks [9,10,11,12,13,14,15] and, on the other side, there was some existing mathematical literature on related processes, such as the Brownian motion on a hyperbolic plane [16,17,18].

We must point out, however, that we are neither replacing the topological space nor assuming that the metric space is determined by the real-world distances between the sites. Consider, for instance, the case of anomalous diffusion [19] in disordered media [20,21]. Quenched disorder may be the result of the interaction of an initially homogeneous medium with an external (random) potential, which produces uneven transition rates between the sites [22,23,24,25,26]. Therefore, the metric space that induces inhomogeneities in the involved probabilities can be the mathematical expression of an energetic landscape.

In this paper, we will extend the analysis done in [8] by considering the case of elliptic geometry, an underlying geometry that forces the topological space to be finite and, thus, equivalent to a ring with two reflecting sites and a forbidden node. This circular arrangement of the nodes should not distract us from the fact that the process describes the evolution of a particle in a linear but finite medium with arbitrary disposition. Indeed, when the number of sites is large, the particle behaves as a homogeneous RW in almost all the domains and experiences the repulsion of the ending points only in their closest vicinity. Therefore, the process can be useful in modeling homogeneous finite and discrete systems surrounded by repulsive fields with limited penetrating power.

The manuscript is structured as follows: In Section 2, we introduce the process and deduce the most general form of the one-step transition probabilities, compatible with the desired loop invariance. We explore the geometric origin of our inhomogeneous probabilities in Section 3, based on stereographic projections of hyperbolic and elliptic metric spaces. We restrict the analysis to the elliptic case from Section 4 on, where we obtain exact and approximate formulas for the probability function of the process and its expected values: the mean and the variance of the position of the walker on the ring. Section 5 is devoted to our analysis of the statistics of extreme events, as first- and last-visit probabilities. Our conclusions are drawn in Section 6, while we leave to the appendix some lengthy mathematical expressions.

## 2. Heterogeneous Processes with Invariant Loop Probabilities

Let us consider the one-dimensional random walk $X_{t}$ in all its generality, a (possibly infinite) Markov chain defined on the integers, i.e., $X_{t}\in Z$ for $t\;\in \;N_{0}$, with $X_{0}\equiv X_{t=0}$ known. The one-step evolution of the process is tied to a set of site-dependent transition probabilities: if, at time *t*, the walker is at a given location, $X_{t}=n$, then at time t+1, one has

(1) $$X_{t+1}=\left\{\begin{array}{cc} n+1, & \mathrm{with}\;\mathrm{probability}\;p_{n\to n+1},\\ n-1, & \mathrm{with}\;\mathrm{probability}\;p_{n\to n-1}, \end{array}\right.$$

with $p_{n\to n+1}+p_{n\to n-1}=1$. We are interested in finding the conditions that yield translationally-invariant loop probabilities, that is, those cases for which

(2) $$p_{n\to n\pm 1}\cdot p_{n\pm 1\to n}=p^{2},$$

with *p* as a constant parameter, 0<p<1, even when $p_{n\to n\pm 1}$ depends on *n*. Note how condition (2) is satisfied in the case of a homogeneous random walk for which p=1/2. Indeed, this value represents a threshold that divides the problem into two well-different domains since one may express *p* as

(3) $$p=\left\{\begin{array}{cc} \frac{1}{2\cosh(\xi )}, & 0<p<\frac{1}{2},\\ \frac{1}{2\cos(\theta )}, & \frac{1}{2}<p<1. \end{array}\right.$$

Note that, while 0<ξ<∞, one must demand that 0<θ<π/3 (These definitions of ξ and θ may seem arbitrary at this point. Beyond being mathematically correct, there is no reason to prioritize them over other proposals. As we will see below, these definitions ease the algebraic treatment and are well adapted to the geometrical interpretation of the problem. Despite this, alternative parameterizations of *p* can still be considered).

The first possibility, that is, the case for which

(4) $$p=\frac{1}{2\cosh(\xi )},$$

has been analyzed in depth in [8]. Observe how Equation (2) leads to

(5) $$p_{n\to n\pm 1}=\frac{1}{2}\frac{\cosh((n\pm 1)\xi )}{\cosh(\xi )\cosh(n\xi )}=\frac{1}{2}\left[1\pm \tanh(\xi )\tanh(n\xi )\right],$$

once symmetry with respect to the origin is demanded: if

(6) $$p_{n\to n\pm 1}=p_{-n\to -n\mp 1},$$

then $p_{0\to \pm 1}=1/2$ necessarily, and the general expression follows. One can easily check that the one-step transition probabilities in (5) generate a process that behaves as a simple symmetric random walk in the vicinity of n=0, and as a *non-reverting*, biased random walk in outer regions of the line. We refer the reader to [8] for further information about the statistical properties of a particle moving according with this infinite Markov chain.

The second scenario, when

(7) $$p=\frac{1}{2\cos(\theta )},$$

is instead more delicate. The one-step probabilities that are derived from Equation (7) with the constraint in (6) read

(8) $$p_{n\to n\pm 1}=\frac{1}{2}\frac{\cos((n\pm 1)\theta )}{\cos(\theta )\cos(n\theta )}=\frac{1}{2}\left[1\mp \tan(\theta )\tan(n\theta )\right],$$

an expression that differs from (5) in a very fundamental aspect: while this formula ensures that $0<p_{n\to n\pm 1}<1$ for any choice of ξ, Equation (8) will finally produce bigger-than-one transition probabilities for a general value of θ, even if 0<θ<π/3. A natural way of avoiding this eventuality is to require that θ be such that cos((n+1)θ)=0 for some value of *n*, i.e., if one has

(9) $$\theta =\frac{\pi }{2(N+1)},$$

with $N\in N_{1}$ (We discard the case N=0, because then no loop can be defined. Even so, some of the expressions below are still valid for this case), then $p_{N\to N+1}=0$ and $p_{N\to N-1}=1$, as well as $p_{-N\to -N-1}=0$ and $p_{-N\to -N+1}=1$. Note that now the possible values of θ are restricted to belong to the range 0<θ<π/4, and n∈{−N,−N+1,…,N−1,N}. Therefore, the process evolves again as an ordinary random walk in the neighborhood of the starting point, and like a *reverting* random walk as the process leaves this region. The strength of the reverting bias increases until it becomes that of a hard wall at positions n=±N.

For clarity reasons, the values of *N* that we are going to use in the illustrative examples to be introduced along the text are relatively small—about ten; this enhances the peculiarities of the process in front of a homogeneous random walk with hard ending points. However, as *N* increases, the *fraction* of sites whose transition probabilities differ significantly from 1/2 decreases as 1/N. This implies, in practice, that our process can be useful in the analysis of homogeneous finite systems bounded by soft walls.

## 3. A Geometric View of the Problem

Before analyzing the properties of this inhomogeneous random walk equipped with reflexing barriers, let us discuss how one can recover the one-step probabilities that drive the dynamics of $X_{t}$ from geometric arguments. Once again, we resort to the idea that, coexisting with the topological structure of the state space of the process, there is an auxiliary metric space that assigns distances to the different locations of the chain and the ratio of the transition probabilities depends inversely on the relative distance between the origin and destination points.

**Figure 1:**
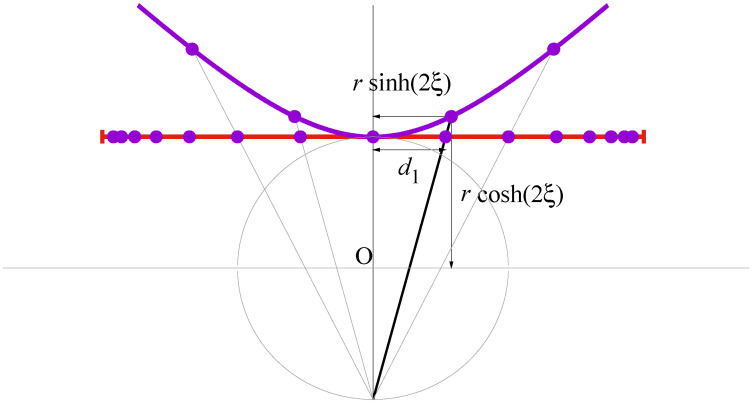
Projection of a hyperbolic geometry. The points at the hyperbola are placed at the positions $x_{n}=r\sinh\left(2n\xi \right)$, $y_{n}=r\cosh\left(2n\xi \right)$, n∈Z. The segment in red corresponds to the stereographic projection of the hyperbola, with the points located at the horizontal positions $d_{n}=2r\tanh\left(n\xi \right)$.

In particular, in [8] we show how one can recover (5) from the distances defined in the absolute of a one-dimensional hyperbolic geometry. Here, we derive anew this result by introducing a minor modification: This time, we analyze the stereographic projection of the hyperbola in which the points in the segment are determined from the intersection between the line that connects the hyperbola centered at the origin with the distal point of a tangential circumference also centered at the origin, see Figure 1.

In [8], the intersecting line did not end at the *south pole* of the circumference but at the origin itself, what defines a gnomonic instead of a stereographic projection. In a one-dimensional problem like ours, the difference reduces to some rescaling of the factors involved that does not affect the essence of discussion, and, in our opinion, stereographic projections provide a smoother transition between both scenarios: the present hyperbolic case and the elliptic one.

If the points at the hyperbola are placed at regular positions $x_{n}=r\sinh\left(2n\xi \right)$, $y_{n}=r\cosh\left(2n\xi \right)$, the corresponding points on the segment are sited at horizontal locations

(10) $$d_{n}\equiv 2r\frac{\sinh(2n\xi )}{1+\cosh(2n\xi )}=2r\tanh\left(n\xi \right),$$

and therefore the $L^{1}$ distance between any two of them is

(11) $$d_{n,m}\equiv \left|d_{n}-d_{m}\right|=2r\frac{\sinh(|n-m|\xi )}{\cosh(n\xi )\cosh(m\xi )}.$$

Then, if we assume that the probability of a one-step transition is inversely proportional to the distance between nearest neighbors,

(12) $$\frac{p_{n\to n+1}}{p_{n\to n-1}}=\frac{d_{n-1,n}}{d_{n,n+1}}=\frac{\cosh((n+1)\xi )}{\cosh((n-1)\xi )},$$

and one recovers Equation (5).

**Figure 2:**
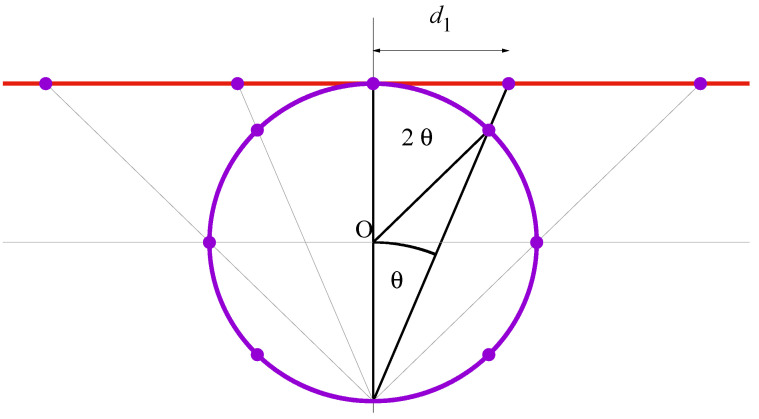
Projection of an elliptic geometry. The points in the circle are placed at regular angular distances, $x_{n}=r\sin$(2n θ), $y_{n}$ = rcos(2n θ), n∈{−N,−N+1,…,N−1,N}. The red line corresponds to the stereographic projection of the circle, with the points sited at locations $d_{n}=2r\tan\left(n\theta \right)$. (N=3 in this figure).

A similar reasoning leads to expression (8), by replacing the hyperbolic geometry by an elliptic geometry: consider a set of 2N+1 points placed in a regular disposition on a circumference of radius *r*: $x_{n}=r\sin\left(2n\theta \right)$, $y_{n}=r\cos\left(2n\theta \right)$, with the values of *n* such that n∈{−N,−N+1,…,N−1,N} and θ defined as in Equation (9). Now consider the stereographic projection of these points into the line tangent to the upper part of the circumference: the projected points are at the intersect between this horizontal line, and the line that connects the original spots and the south pole of the circumference, where there is no accessible point, see Figure 2.

The projected points on the horizontal line are sited at positions marked by

(13) $$d_{n}\equiv 2r\frac{\sin(2n\theta )}{1+\cos(2n\theta )}=2r\tan\left(n\theta \right),$$

and the $L^{1}$ distance between them reads

(14) $$d_{n,m}\equiv \left|d_{n}-d_{m}\right|=2r\frac{\sin(|n-m|\theta )}{\cos(n\theta )\cos(m\theta )}.$$

If we demand the same kind of interdependence between transition probabilities and distances as in (12), i.e.,

(15) $$\frac{p_{n\to n+1}}{p_{n\to n-1}}=\frac{d_{n-1,n}}{d_{n,n+1}}=\frac{\cos((n+1)\theta )}{\cos((n-1)\theta )},$$

one recovers Equation (8) with the understanding that we assign $d_{\pm (N+1)}=\pm \infty$ to these inaccesible points: otherwise one cannot formally recover $p_{\pm N\to \pm (N+1)}=0$. Note that this is consistent with Equation (13) as well as with the fact that

(16) $$d_{\pm N}=\pm 2r\tan\left(\frac{\pi }{2}\cdot \frac{N}{N+1}\right)\underset{N\to \infty }{\to }\pm \infty ,$$

since, in the original elliptic geometry, both values n=±(N+1) would be coincident with the south pole of the circumference. Indeed, as we will see below, it is operationally convenient to include ±(N+1) within the spectrum of values of $X_{t}$, by attaching a null probability to these events.

Finally, we observe how site-to-site distances increase as |n| increases. This behavior is in clear contrast with the hyperbolic case where distances decrease with |n|, because, in the latter, the length of the projective segment is finite. The borderline case corresponds to the (flat) Euclidean geometry, which can be recovered by letting ξ,θ→0, and r→∞ in such a way that their product remains finite.

## 4. Probability Functions

Once we have discussed the geometric interpretation of both processes, we will focus our attention on the statistical properties of the elliptic setup, as the hyperbolic case was analyzed in [8]. We will begin with the probability function $p_{n,t}$, the probability of finding the process at site *n* at time *t*, if it started from the origin:

(17) $$p_{n,t}\equiv P\left(X_{t}=n|X_{0}=0\right),$$

for n∈{−N−1,−N,…,N,N+1}, with the proviso that $p_{-N-1,t}=p_{N+1,t}=0$ for any value of *t*. Here and hereafter, we denote by $P\left(\cdot \right)$ the probability of its argument.

Let us consider, in the first place, the case in which t≤N and assume for the moment that n>0. In order to have $p_{n,t}\neq 0$, we need to demand, on the one hand, that *n* and *t* have the same parity (i.e., if both are odd or even integers), and, on the other hand, that n≤t, otherwise the site *n* is inaccessible. The transition probability $p_{0\to n,t}$ of a path connecting sites 0 and *n* in *t* steps can be expressed under these circumstances as

(18) $$\begin{array}{ccc} p_{0\to n,t} & = & p_{0\to 1}\cdots p_{n-1\to n}\cdot p^{t-n}=\frac{\cos(n\theta )}{{\left[2\cos(\theta )\right]}^{n}}\cdot \frac{1}{{\left[2\cos(\theta )\right]}^{t-n}}\\ & = & \frac{\cos(n\theta )}{{\left[2\cos(\theta )\right]}^{t}}, \end{array}$$

where we have used (2), (7) and (8). As it can be observed, Equation (18) does not depend on the particular path followed, and therefore $p_{n,t}$ is $p_{0\to n,t}$ times the number of different paths that go from 0 to *n* in *t* steps, a quantity that can be computed by resorting to standard combinatorial arguments, leading to

(19) pn,t=tt−n2cos(nθ)2cos(θ)t.

Note how $p_{-n,t}=p_{n,t}$, which implies that Equation (19) is indeed valid for |n|≤t≤N, if *n* and *t* have the same parity:

(20) pn,t=tt−n2cos(nθ)2cos(θ)t1t−|n|2∈N0,

where $1_{A}$ is equal to one if *A* is true and zero otherwise.

The condition t≤N eases the counting problem, since, for t>N, we will have to subtract, from the binomial term, the *forbidden* paths on Z, those paths that connect 0 and *n* going through site N+1 and/or −N−1. Instead of following this route at this point, let us consider the recursion relation that satisfies $p_{n,t}$,

(21) $$\begin{array}{ccc} p_{n,t} & = & p_{n-1,t-1}\cdot p_{n-1\to n}+p_{n+1,t-1}\cdot p_{n+1\to n}\\ & = & \frac{1}{2}\frac{p_{n-1,t-1}\cdot \cos\left(n\theta \right)}{\cos((n-1)\theta )\cos(\theta )}+\frac{1}{2}\frac{p_{n+1,t-1}\cdot \cos\left(n\theta \right)}{\cos((n+1)\theta )\cos(\theta )}, \end{array}$$

an expression that can be rewritten as

(22) $$\begin{array}{ccc} & & p_{n,t}\left[e^{i2n\theta }+e^{-i2n\theta }+2\cos\left(2\theta \right)\right]\cos\left(\theta \right)\\ & = & p_{n-1,t-1}\left[e^{i(2n+1)\theta }+e^{-i(2n+1)\theta }+2\cos\left(\theta \right)\right]/2\\ & + & p_{n+1,t-1}\left[e^{i(2n-1)\theta }+e^{-i(2n-1)\theta }+2\cos\left(\theta \right)\right]/2. \end{array}$$

The problem posed can be solved with the use of Discrete Fourier Transform (DFT) pairs

(23) $$\begin{array}{ccc} {\tilde{u}}_{k} & \equiv & \sum_{n=-N}^{N+1}u_{n}\cdot e^{-i\pi kn/(N+1)}=\sum_{n=-N}^{N+1}u_{n}\cdot e^{-i2kn\theta }, \end{array}$$

(24) $$\begin{array}{ccc} u_{n} & \equiv & \frac{1}{2(N+1)}\sum_{k=-N}^{N+1}{\tilde{u}}_{k}\cdot e^{i\pi kn/(N+1)}=\frac{1}{2(N+1)}\sum_{k=-N}^{N+1}{\tilde{u}}_{k}\cdot e^{i2kn\theta }, \end{array}$$

definitions that have embedded the following property of periodicity: $u_{n+2\ell (N+1)}=u_{n}$, ${\tilde{u}}_{k+2\ell (N+1)}={\tilde{u}}_{k}$, for ℓ∈Z. Therefore, the sums in Equations (23) and (24) could alternatively have begun at −N−1 and ended at *N*, but we cannot include contemporarily the terms corresponding to −N−1 and N+1, even though, in our case, $p_{n,t}=0$ for |n|=N+1. In fact, we must proceed with caution, since we have $p_{n,t}=0$ for |n|≥N+1, a boundary condition that is assumed in (22) (In addition, $p_{n,t}=0$ if *n* and *t* have different parity, but this fact does not affect the present discussion).

To find the solution to Equation (22), let us multiply the whole expression by $e^{-i2kn\theta }$, sum from *n* equal to −N to *N*, take into account the boundary conditions where they apply, and obtain

(25) $$\begin{array}{ccc} & & \left[{\tilde{p}}_{k-1,t}+{\tilde{p}}_{k+1,t}+2\cos\left(2\theta \right){\tilde{p}}_{k,t}\right]\cos\left(\theta \right)\\ & = & \cos\left((2k-3)\theta \right){\tilde{p}}_{k-1,t-1}+\cos\left((2k+3)\theta \right){\tilde{p}}_{k+1,t-1}\\ & + & 2\cos\left(\theta \right)\cos\left(2k\theta \right){\tilde{p}}_{k,t-1}. \end{array}$$

As in the case of Equation (21), we could use this recursion in conjunction with the fact that ${\tilde{p}}_{k,0}=1$ to compute ${\tilde{p}}_{k,t}$. Since $p_{n,t}$ is real and symmetric, so is ${\tilde{p}}_{k,t}$. Moreover, as ${\tilde{p}}_{k,t}$ is a probability function, ${\tilde{p}}_{0,t}=1$. Therefore, we can compute ${\tilde{p}}_{\pm 1,1}$ from (25)

(26) $${\tilde{p}}_{\pm 1,1}=\frac{\cos(3\theta )}{\cos(\theta )}+1-\cos\left(2\theta \right)=1-2\sin^{2}\left(\theta \right),$$

and then

(27) $${\tilde{p}}_{k+1,1}=\frac{\cos\left((2k-3)\theta \right)+\cos\left((2k+3)\theta \right)}{\cos(\theta )}+2\cos\left(2k\theta \right)-{\tilde{p}}_{k-1,1}-2\cos\left(2\theta \right){\tilde{p}}_{k,1},$$

for k>0. Once we have found all the ${\tilde{p}}_{k,1}$, we can proceed similarly with ${\tilde{p}}_{k,2}$ and so on.

Fortunately, we can obtain a closed form for ${\tilde{p}}_{k,t}$. From (20) we have that, for t≤N,

(28) p˜k,t=∑n=−NN+1pn,t·e−i2knθ=∑n=−tttt−n2cos(nθ)2cos(θ)t1t−|n|2∈N0·e−i2knθ.

This means that

(29) p˜k,t=∑j=0ttjcos((2j−t)θ)2cos(θ)t·e+i2k(2j−t)θ=122cos(θ)t∑j=0ttje+i(2k+1)(2j−t)θ+e−i(2k−1)(2j−t)θ=12cos((2k+1)θ)cos(θ)t+12cos((2k−1)θ)cos(θ)t.

Note that, in particular, ${\tilde{p}}_{0,t}=1$ and

(30) $${\tilde{p}}_{\pm 1,t}=\frac{1}{2}+\frac{1}{2}{\left[\frac{\cos(3\theta )}{\cos(\theta )}\right]}^{t}=\frac{1}{2}+\frac{1}{2}{\left[1-4\sin^{2}\left(\theta \right)\right]}^{t},$$

in concordance with the result found in (26). The point is that expression (29) satisfies relation (25), for any value of *t* and θ, and thus it is the general solution for ${\tilde{p}}_{k,t}$.

By no means does this imply that $p_{n,t}$ can be expressed as in Equation (20) for t>N; we must invert (29) to obtain

(31) pn,t=cos(nθ)2cos(θ)t∑ℓ=ℓminℓmaxtt−n2−ℓ(N+1)(−1)ℓ1t−|n|2∈N0,

with

(32) $$\begin{array}{ccc} \ell_{\min} & = & \left\lceil -\frac{t+n}{2(N+1)}\right\rceil , \end{array}$$

(33) $$\begin{array}{ccc} \ell_{\max} & = & \left\lfloor \frac{t-n}{2(N+1)}\right\rfloor , \end{array}$$

and where ⌈·⌉ and ⌊·⌋ are the ceiling function and floor function, respectively.

**Figure 3:**
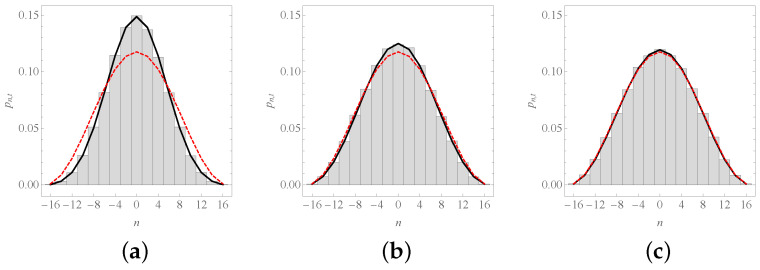
The probability function $p_{n,t}$. We depict the probability of finding the system at position *n* after: (**a**) t=40 steps; (**b**) t=80 steps; and (**c**) t=120 steps; if $X_{0}=0$ and N=16. As the values of *t* are even quantities, only even values of *n* are shown. The solid curve corresponds to Equation (31), the red dashed curve to Equation (34), and histograms were obtained from 100,000 numerical simulations of the process, with the binning (here and hereafter) chosen to include only one attainable site in each category.

Figure 3 shows three snapshots of the time evolution of $p_{n,t}$ for N=16. Note how, for both instances, the theoretical curve and the histogram converge to the dashed curve. This curve represents the quasi-steady-state probability function $p_{n}^{\mathrm{even}}$,

(34) $$p_{n}^{\mathrm{even}}=\frac{2}{N+1}\cos^{2}\left(n\theta \right)1_{\frac{n}{2}\in Z},$$

to which the probability function tends. The reason for calling this a quasi-steady state is because the graph is bipartite: starting from the origin, the particle can be found in odd locations if and only if *t* is odd, and in even locations if and only if *t* is even. Therefore, Equation (34) is valid when *t* is even. Fortunately, the steady state when *t* (and *n*) is odd is just the same

(35) $$p_{n}^{\mathrm{odd}}=\frac{2}{N+1}\cos^{2}\left(n\theta \right)1_{\frac{n-1}{2}\in Z},$$

and, since these two scenarios have the same likelihood due to the alternation of even and odd values of *t*, one arrives at

(36) $$p_{n}^{\mathrm{eq}.}=\frac{1}{2}p_{n}^{\mathrm{odd}}+\frac{1}{2}p_{n}^{\mathrm{even}}=\frac{1}{N+1}\cos^{2}\left(n\theta \right),$$

the equilibrium distribution in the ergodic sense. The very existence of this steady-state and the expression itself can be deduced from (29). Let ${\tilde{p}}_{k}$ be

(37) $$\begin{array}{ccc} {\tilde{p}}_{k} & \equiv & \lim_{t\to \infty }{\tilde{p}}_{k,t}=\lim_{t\to \infty }\frac{1}{2}{\left[\frac{\cos((2k+1)\theta )}{\cos(\theta )}\right]}^{t}+\frac{1}{2}{\left[\frac{\cos((2k-1)\theta )}{\cos(\theta )}\right]}^{t}\\ & = & \frac{1}{2}\delta_{k,-1}+\delta_{k,0}+\frac{1}{2}\delta_{k,1}, \end{array}$$

where $\delta_{k,\ell }$ is the Kronecker delta that returns 1 if k=ℓ and zero otherwise. The inversion of (37) leads to (36).

Let us consider now the counting-path approach. To this end, let us express Equation (31) in the following form

(38) pn,t=cos(nθ)2cos(θ)t∑ℓ=−∞∞tt−(−1)ℓn2−ℓ(N+1)(−1)ℓ1t−|n|2∈N0,

with the understanding that

(39) ab=0,

if either *b* or b−a are negative integers. The most noticeable change in this formula is the replacement $n\mapsto {\left(-1\right)}^{\ell }n$. This can be understood on the basis of mirroring arguments: For N<t<3(N+1), the number of paths beginning at 0 and ending at *n* touching or passing though N+1 is equal to the number of trajectories starting at 0 and reaching the point 2(N+1)−n,

tt−2(N+1)+n2=tt+n2−(N+1)=tt−n2+(N+1),

if *t* and *n* have the same parity. These paths must be subtracted from the total, as well as the trajectories beginning at 0 and ending at *n* touching or passing through −N−1,

tt+2(N+1)+n2=tt+n2+(N+1)=tt−n2−(N+1).

Therefore, when the starting point is the origin, the change of sign introduced by every refection can be dropped after a rearrangement of the terms.

This is no longer true if m≠0. In such a case

(40) pn,t;m=cos(nθ)cos(mθ)2cos(θ)t∑ℓ=−∞∞tt−(−1)ℓn+m2−ℓ(N+1)(−1)ℓ1t−|n−m|2∈N0,

that is, for t≥|m−n| and sharing both magnitudes the same parity, one has

(41) $$\begin{array}{c} p_{n,t;m}=\frac{\cos(n\theta )}{\cos\left(m\theta \right){\left[2\cos(\theta )\right]}^{t}}C_{n,t;m}^{\left[-N,N\right]}, \end{array}$$

where the leading factor is just $p_{m\to n,t}$, the product of transition probabilities of a path connecting sites *m* and *n* in *t* steps, and

(42) Cn,t;m[L,M]≡∑ℓ=−∞∞tt−(−1)ℓn−M+L2+(m−M+L2)−ℓM−L+22(−1)ℓ1t−|n−m|2∈N0

is the number of paths stating at $X_{0}=m$ and ending at $X_{t}=n$, in such a way that one has that $L\leq X_{t^{\prime }}\leq M$, $0\leq t^{\prime }\leq t$. Note that the properties of binomials guarantee that factor ${\left(-1\right)}^{\ell }$ can be moved from the parenthesized term with the *n* to the one with the *m* after some reordering, as demanded by the “time reversal” symmetry of this magnitude,

(43) $$C_{n,t;m}^{\left[L,M\right]}=C_{m,t;n}^{\left[L,M\right]}.$$

**Figure 4:**
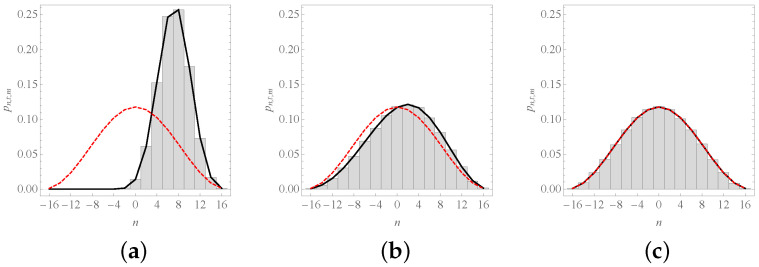
The probability function $p_{n,t;m}$. We depict the probability of finding the system at position *n* after: (**a**) t=10 steps; (**b**) t=150 steps; and (**c**) t=1000 steps; if m=8 and N=16. As the values of *t* are even quantities, only even values of *n* are shown. The solid curve corresponds to Equation (40), the red dashed curve to Equation (36), and histograms were obtained from 100,000 numerical simulations of the process.

In practical implementations of Formula (40), as in the confection of Figure 4, one can replace the limits in (40) with ±L,

(44) $$\begin{array}{ccc} L & \equiv & \left\lfloor \frac{t+|n|+|m|}{2(N+1)}\right\rfloor . \end{array}$$

This is a good proxy since, at most, one must discard two terms in the sum. A more precise expression leads to a piece-wise definition of $p_{n,t;m}$ that is highly dependent on the particular values of *N*, *n*, *m*, and *t*, see Appendix A.

The intricacy of Equation (40) discourages the search for a general expression for the expected value, $E\left[\cdot \right]$, of the position

(45) $$\begin{array}{c} \mu_{t;m}\equiv E\left[X_{t}|X_{0}=m\right]=\sum_{n=m-t}^{m+t}n\cdot p_{n,t;m}, \end{array}$$

although some approximations can be considered. For t≤N−|m|, one has

(46) μt;m≃∑k=0ttkm−2k+tcos(mθ)cos((m−2k+t)θ)2cos(θ)t=m−tan(θ)tan(mθ)·t,

the process shows reversion to the origin, and ultimately

(47) $$\begin{array}{c} \lim_{t\to \infty }\mu_{t;m}=0, \end{array}$$

from the existence of the equilibrium probability (40). From this same expression, one can conclude that the time evolution of the standard deviation of the process, $\sigma_{t;m}$,

(48) $$\begin{array}{ccc} \sigma_{t;m}^{2} & \equiv & E\left[X_{t}^{2}|X_{0}=m\right]-\mu_{t;m}^{2}, \end{array}$$

will attain a limiting value as well,

(49) $$\begin{array}{c} \lim_{t\to \infty }\sigma_{t;m}^{2}=\sum_{n=-N}^{N}n^{2}\cdot \frac{1}{N+1}\cos^{2}\left(n\theta \right)=\frac{1}{6}\left(2N^{2}+4N+3\right)-\frac{1}{2\sin^{2}\left(\theta \right)}, \end{array}$$

while, for small values of *t*, one has

(50) σt;m2≃∑k=0ttk(m+2k−t)2cos(mθ)cos((m+2k−t)θ)2cos(θ)t−m−tan(θ)tan(mθ)t2=1cos2(θ)t−tan2(θ)t2−tan2(θ)tan2(mθ)t2=1cos2(θ)t−tan2(θ)cos2(mθ)t2.

**Figure 5:**
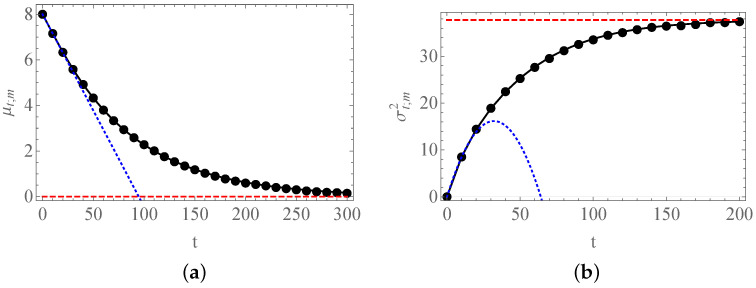
Expected values of $p_{n,t;m}$. In (**a**), we consider the evolution of $\mu_{t;m}$ for m=8. The solid black curve is the exact evolution predicted by $p_{n,t;m}$, Equation (45), the blue dotted curve depicts the linear behavior predicted by Equation (46), and the red dashed line coincides with the origin. In (**b**), we show the bounded growth $\sigma_{t;m}^{2}$, for m=8. Again, the solid black curve represents the exact Formula (48), the blue dotted curve corresponds to approximate expression (50), while the red dashed line stems from Equation (49). In both cases, N=16, and the solid circles were obtained from 100,000 numerical simulations of the process.

We can observe in Figure 5 the good agreement between the numerical simulations of the process and the different expressions found: In Figure 5a, we observe the slow return of the mean of the process to the origin, whereas in Figure 5b, we find how the variance of the process exhibits a sigmoid-like shape, tending toward the value dictated by the equilibrium probability. As we will see in the next section, the exponential character of the time-evolution of the process is behind these features.

## 5. First- and Last-Time Events

Equation (40) can be easily generalized to $p_{n,t;m}^{\left[L,M\right]}$,

(51) $$p_{n,t;m}^{\left[L,M\right]}\equiv P\left(\left.X_{t}=n\right|X_{0}=m,L\leq X_{t^{\prime }}\leq M,0\leq t^{\prime }\leq t\right),$$

the probability that the process $X_{t}$ goes form $X_{0}=m$ to $X_{t}=n$, in such a way that one has that $L\leq X_{t^{\prime }}\leq M$, $0\leq t^{\prime }\leq t$, due to the factorization of this probability as the likelihood of a single path connecting points *m* and *n* in *t* steps (which is always the same irrespective of the trajectory) times the amount of those trajectories that do not exceed the chosen limits, $C_{n,t;m}^{\left[L,M\right]}$, see Equation (42),

(52) $$p_{n,t;m}^{\left[L,M\right]}=\frac{\cos(n\theta )}{\cos\left(m\theta \right){\left[2\cos(\theta )\right]}^{t}}C_{n,t;m}^{\left[L,M\right]}.$$

This quantity can be used to obtain the survival probability, $S_{t;m}^{\left[L,M\right]}$, the probability that, at time *t*, the process starting from $X_{0}=m$ has never left the interval [L,M],

(53) $$S_{t;m}^{\left[L,M\right]}=\sum_{n=L}^{M}p_{n,t;m}^{\left[L,M\right]}.$$

Please, note that the only restriction in the parameter set affecting the survival probability is that we denoted by *L* the lower limit of the interval and by *M* its higher limit, L≤M. Thus, in this case, $S_{t;m}^{\left[L,M\right]}$ is well defined having *t* and *m* with either the same parity or not.

Survival probabilities are commonly used to compute the probability that the first-visit of the process to site *n*, starting from site *m*, has taken place at time *t*,

(54) $$f_{t,n;m}\equiv P\left(T_{n;m}=t\right),$$

where the random variable $T_{n;m}$ is defined as [3]

(55) $$T_{n;m}\equiv \min\left\{t>0:X_{t}=n|X_{0}=m\right\},$$

through the relationship

(56) $$f_{t,n;m}=S_{t-1;m}^{\left[-N,n-1\right]}-S_{t;m}^{\left[-N,n-1\right]},$$

for −N≤m<n≤N, and the relationship

(57) $$f_{t,n;m}=S_{t-1;m}^{\left[n+1,N\right]}-S_{t;m}^{\left[n+1,N\right]},$$

for −N≤n<m≤N. In the present case, these expressions are very intricate to be used in practice due to the abundance of summations, and the following alternative procedure leads to more compact expressions: Consider, in the first place, a configuration that satisfies −N≤m<n≤N, then the only way of reaching *n* at time *t* for the first time, is being at n−1 by t−1, without having surpassed this point previously, and a final transition n−1→n,

(58) $$\begin{array}{ccc} f_{t,n;m} & = & p_{n-1,t-1;m}^{\left[-N,n-1\right]}\times p_{n-1\to n}=\frac{\cos(n\theta )}{\cos\left(m\theta \right){\left[2\cos(\theta )\right]}^{t}}C_{n-1,t-1;m}^{\left[-N,n-1\right]} \end{array}$$

where the typical restrictions to values and parities of *n*, *m*, and *t* considered throughout the text do apply here. If one has that −N≤n<m≤N, then

(59) $$\begin{array}{ccc} f_{t,n;m} & = & p_{n+1,t-1;m}^{\left[n+1,N\right]}\times p_{n+1\to n}=\frac{\cos(n\theta )}{\cos\left(m\theta \right){\left[2\cos(\theta )\right]}^{t}}C_{n+1,t-1;m}^{\left[n+1,N\right]}. \end{array}$$

**Figure 6:**
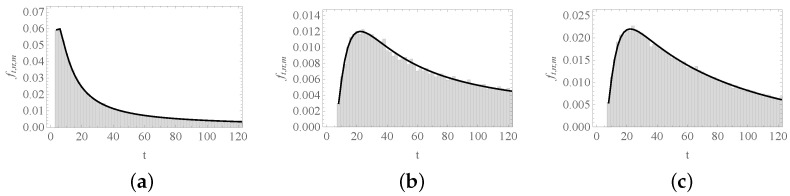
Probability function $f_{t,n;m}$. We depict the probability of that the first visit of the process to site *n* starting from *m* occurs after *t* steps: (**a**) n=4 and m=0; (**b**) n=8 and m=0; and (**c**) n=0 and m=8. In all cases N=16. As n−m is even, only even values of *t* are shown. The solid curve corresponds to Equation (58) or (59), and histograms were obtained from 100,000 numerical simulations of the process.

Figure 6 shows three instances of $f_{t,n;m}$, for different choices of *n* and *m*.

In fact, this approach paves the way for the computation of $f_{2t,n;n}$, the probability that the process *returns* for the first time to a given point *n* after 2t steps, $f_{2t,n;n}$,

(60) $$\begin{array}{ccc} f_{2t,n;n} & = & p_{n\to n-1}\times p_{n-1,2t-2;n-1}^{\left[-N,n-1\right]}\times p_{n-1\to n}+p_{n\to n+1}\times p_{n+1,2t-2;n+1}^{\left[n+1,N\right]}\times p_{n+1\to n}\\ & = & \frac{1}{{\left[2\cos(\theta )\right]}^{2t}}\left(C_{n-1,2t-2;n-1}^{\left[-N,n-1\right]}+C_{n+1,2t-2;n+1}^{\left[n+1,N\right]}\right). \end{array}$$

When t≤N−|n|, the first-return probability does not depend on *n* and satisfies a well-known identity for regular random walks,

(61) f2t,n;n=12t−112cos(θ)2t2tt=12t−1pn,2t;n,

but this formula ceases to be valid as *t* grows. On the one side, as we have already seen, $p_{n,2t;n}$ tends to a time-independent function of *n*, see Equations (34) and (35),

$$p_{n,2t;n}≃\frac{2}{N+1}\cos^{2}\left(n\theta \right),$$

and, on the other side, $f_{2t,n;n}$ decays exponentially with *t*, as we will prove next. To this end, we have to analyze $C_{n,t;m}^{\left[L,M\right]}$ for t≫M−L. Indeed, from very existence of the steady-state distribution and using symmetry arguments, one concludes that we can approximate this magnitude by the following expression:

(62) $$C_{n,t;m}^{\left[L,M\right]}≃\frac{4}{M-L+2}\cos\left(\frac{\pi }{2}\frac{2n-M-L}{M-L+2}\right)\cos\left(\frac{\pi }{2}\frac{2m-M-L}{M-L+2}\right){\left[2\cos\left(\frac{\pi }{M-L+2}\right)\right]}^{t}.$$

According to Equation (60), we need to evaluate

(63) $$\begin{array}{ccc} C_{n-1,t;n-1}^{\left[-N,n-1\right]} & ≃ & \frac{4}{N+n+1}\sin^{2}\left(\frac{\pi }{N+n+1}\right){\left[2\cos\left(\frac{\pi }{N+n+1}\right)\right]}^{t}, \end{array}$$

and

(64) $$\begin{array}{ccc} C_{n+1,t;n+1}^{\left[n+1,N\right]} & ≃ & \frac{4}{N-n+1}\sin^{2}\left(\frac{\pi }{N-n+1}\right){\left[2\cos\left(\frac{\pi }{N-n+1}\right)\right]}^{t}, \end{array}$$

to assess the behavior of $f_{2t,n;n}$ for large values of *t*. If n=0, Equations (63) and (64) are coincident, and we arrive to the following compact expression:

(65) $$\begin{array}{c} f_{2t,0;0}≃\frac{2}{N+1}\tan^{2}\left(2\theta \right){\left[\frac{\cos\left(2\theta \right)}{\cos\left(\theta \right)}\right]}^{2t}. \end{array}$$

In general, the most stringent term is the one for which N±n+1 is smaller, i.e.,

(66) $$\begin{array}{c} f_{2t,n;n}≃\frac{1}{N-|n|+1}\tan^{2}\left(\frac{\pi }{N-|n|+1}\right){\left[\frac{\cos\left(\frac{\pi }{N-|n|+1}\right)}{\cos\left(\theta \right)}\right]}^{t}. \end{array}$$

**Figure 7:**
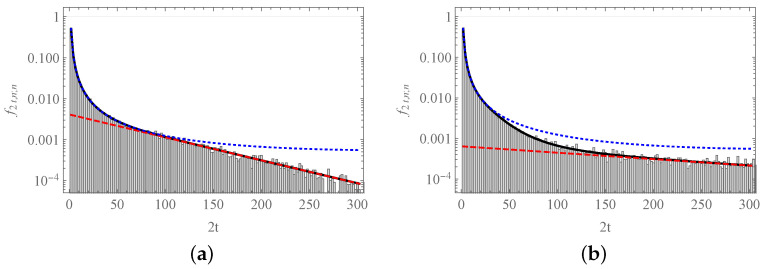
The probability function $f_{2t,n;n}$. We depict the probability of that the first return of the process to site *n* occurs after 2t steps: (**a**) n=0; (**b**) n=8. In both cases, N=16. The solid curve corresponds to Equation (60), the blue dotted curve depicts Equation (61), and the red dashed lines correspond to Equations (65) and (66), respectively. Histograms were obtained from 100,000 numerical simulations of the process.

In Figure 7, we can observe how the approximate formulas are still valid for values of *t* well apart from those used in their respective derivations. In Figure 7a, there is an effective crossover between both limiting behaviors that takes place at t≈40, beyond the restriction t≤N−|n|, as n=0 and N=16. This means that, for any value of *t*, one has a surrogate, compact expression for $f_{2t,0;0}$. In Figure 7b, we find that (61) is still valid outside the region where t≤N−|n|, n=8 now; however, there is no intersection between the expressions (61) and (66). This is due to the presence of the second, transitory exponential regime that dominates the evolution for values of *t* in the range 20≲t≲40.

Consider now $g_{2t,n;2T,n}$, the probability that the *last* return of process to the initial point *n* after 2T steps takes place at time 2t. This probability can be obtained from the likelihood of being in the initial spot at time 2t multiplied by the probability of staying either above of below this value for the remaining period,

(67) $$g_{2t,n;2T,n}=p_{n,2t;n}\left(p_{n\to n-1}\times S_{2(T-t)-1;n-1}^{\left[-N,n-1\right]}+p_{n\to n+1}\times S_{2(T-t)-1;n+1}^{\left[n+1,N\right]}\right).$$

with

$$\begin{array}{ccc} S_{2(T-t)-1;n-1}^{\left[-N,n-1\right]} & = & \sum_{m=-N}^{n-1}p_{m,2(T-t)-1;n-1}^{\left[-N,n-1\right]}\\ & = & \frac{1}{\cos\left(\left(n-1\right)\theta \right){\left[2\cos(\theta )\right]}^{2(T-t)-1}}\sum_{m=-N}^{n-1}\cos\left(m\theta \right)C_{m,2(T-t)-1;n-1}^{\left[-N,n-1\right]}, \end{array}$$

and similarly

$$\begin{array}{ccc} S_{2(T-t)-1;n+1}^{\left[n+1,N\right]} & = & \sum_{m=n+1}^{N}p_{m,2(T-t)-1;n-1}^{\left[n+1,N\right]}\\ & = & \frac{1}{\cos\left(\left(n+1\right)\theta \right){\left[2\cos(\theta )\right]}^{2(T-t)-1}}\sum_{m=n+1}^{N}\cos\left(m\theta \right)C_{m,2(T-t)-1;n+1}^{\left[n+1,N\right]}; \end{array}$$

with the understanding that the whole term inside the parentheses reduces to one if t=T.

In this case, due to the nature of Equation (67), to obtain an approximate formula, one must use contemporarily small and large *t* approximations. Let us focus exclusively on the case n=0 to reduce the mathematical complexity, and consider in the first place that t≤N≪T. For t≤N, we can express the leading factor as

p0,2t;0=12cos(θ)2t2tt,

whereas for T−t≫N, we have

$$\begin{array}{c} C_{m,2(T-t)-1;1}^{\left[1,N\right]}=C_{-m,2(T-t)-1;-1}^{\left[-N,-1\right]}≃\frac{4}{N+1}\sin\left(2\theta \right)\sin\left(2m\theta \right){\left[2\sin\left(2\theta \right)\right]}^{2(T-t)-1}, \end{array}$$

for m∈{2,4,⋯,2⌊N/2⌋}. This symmetry leads to

(68) g2t,n;2T,n≃8N+12tt2cos(2θ)2(T−t)−12cos(θ)2Tsin(2θ)∑k=1N/2cos(2kθ)sin(4kθ)=2N+12tt2cos(2θ)2(T−t)2cos(θ)2Tsin2(2θ)sin(θ)sin(3θ),

where we have further assumed that *N* is an even magnitude to avoid the floor function. The reverse situation corresponds to t≫N≥T−t. Here, we have that the task is simpler if we consider the following alternative expression for $g_{2t,n;2T,n}$,

(69) $$g_{2t,n;2T,n}=p_{n,2t;n}\sum_{t^{\prime }=T-t+1}^{\infty }f_{2t^{\prime },n;n},$$

that is, the probability of being in the initial site at time 2t multiplied by the probability that the next return to this location takes longer than the remaining period. Indeed, to perform the desired analysis, it is better to express (69) as

(70) $$g_{2t,n;2T,n}=p_{n,2t;n}\left(1-\sum_{t^{\prime }=1}^{T-t}f_{2t^{\prime },n;n}\right),$$

since we already know that

f2t′,n;n=12t′−112cos(θ)2t′2t′t′,

for $t^{\prime }\leq N$. Then, if we combine this expression with

$$p_{0,2t;0}≃\frac{2}{N+1},$$

t≫N, we obtain

(71) g2t,0;2T,0≃2N+11−∑t′=1T−t12t′−112cos(θ)2t′2t′t′.

**Figure 8:**
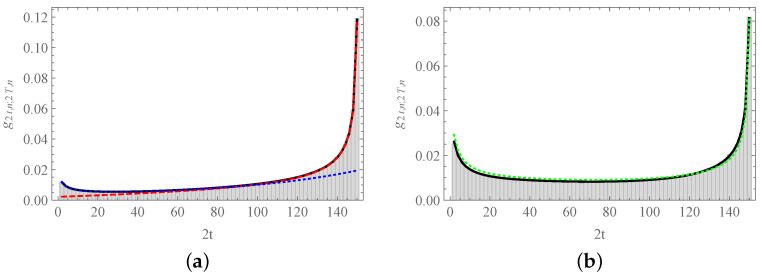
The probability function $g_{2t,n;2T,n}$. We depict the probability that the last return of the process to site *n* after 2T steps occur at time 2t: (**a**) n=0 and (**b**) n=8. In both cases, T=75 and N=16. The solid curve corresponds to Equation (67) in both panels. In panel (**a**), the blue dotted curve shows Equation (68), and the red dashed line corresponds to the approximate Equation (71). In panel (**b**), the green dotted line follows the heuristic Equation (72). Histograms were obtained from 100,000 numerical simulations of the process.

As T−t≤N, the sum contains a reduced number of terms, and it can be formally expressed in terms of the Gaussian hypergeometric function (Note in particular that the sum does not converge for T−t→∞). In Figure 8a, we can observe the goodness of both approximate formulas which, one more time, intersect at t≈40.

Equation (71) can be easily extended to encompass arbitrary values of *n*,

g2t,n;2T,n=2N+1cos2(nθ)1−∑t′=1T−t12t′−112cos(θ)2t′2t′t′,

but we must be aware that *T* must be long enough to allow the system to reach the steady state, which is not the case if N=16 and T=75, cf. Figure 4b. However, for these intermediate situations, heuristic expressions with a clear inspiration in the arcsin law of regular random walks like

(72) $$g_{2t,n;2T,n}≃\frac{1}{\sqrt{4(T-t)+1}}p_{n,2t;n}$$

may become useful, see Figure 8b.

## 6. Conclusions and Future Work

The random walks with invariant loop probabilities constitute a family of Markov processes which, having site-dependent transition probabilities, can be addressed using simple analytical tools. This new class of stochastic processes splits in two different subclasses delimited by the simple random walk in one dimension; however, despite that, the complete family can be obtained by resorting to a single geometrical argument—the stereographic projection of an underlying metric space.

The case in which this associated metric space leads to hyperbolic probabilities was analyzed in depth in a previous work. Here, we concentrated our efforts in the complementary case, the stereographic projection of a circle into the real line that defines a set of elliptic probabilities. The formalism induces the automatic emergence of two ending, reflexing nodes in the ring that surrounds a forbidden site with a regular disposition of the rest of the nodes.

We derived the probability function of the process in the first place, a function that tends to a steady-state distribution. After that, we considered the statistical properties related to extreme events: the waiting time until the first visit to a target if the process is presently at some given location, or the probability of having witnessed the last visit to a target if the observation time is finite.

We leave, for future work, the search for physical implementations of the underlying metric space that can account for the transformation of a regular layout into an inhomogeneous medium whose properties can be satisfactorily captured by one of these stereographic random walks.

Another interesting extension of this model, also left for future publications, consists in the concatenation of finite chains (of different lengths) by their ending points, a setup that may describe a series of basins of attraction. In this case, the final nodes must be partially reflexing and partially transmitting sites, which will break the exact loop invariance assumed here and have a possible impact on the ergodicity of the system.

## Data Availability

The data generated in this study are available on request from the author.

## Abbreviations

The following abbreviations are used in this manuscript:

UB	Universitat de Barcelona
UBICS	Universitat de Barcelona Institute of Complex Systems
RW	Random walk
DFT	Discrete Fourier Transform
AGAUR	Agència de Gestió d’Ajuts Universitaris i de Recerca
AEI	Agencia Estatal de Investigación
FEDER	Fondo Europeo de Desarrollo Regional
UE	Unión Europea

## Appendix A

Assume that *N*, *n*, *m*, and *t* are even quantities, and that 2≤m≤N−2,

(A1) $$p_{n,t;m}=\frac{\cos(n\theta )}{\cos\left(m\theta \right){\left[2\cos(\theta )\right]}^{t}}C_{n,t;m}^{\left[-N,N\right]}.$$

Then, for 0≤t≤N−m one has

Cn,t;m[−N,N]=0,−N≤n≤m−t−2,tt−n+m2,m−t≤n≤m+t,0,m+t+2≤n≤N,

where the last condition cannot be satisfied for t=N−m. For N−m+2≤t≤N+m, one has

Cn,t;m[−N,N]=0,−N≤n≤m−t−2,tt−n+m2,m−t≤n≤2N−m−t,tt−n+m2−tt+n+m2−(N+1),2(N+1)−m−t≤n≤N,

where the first condition cannot be satisfied for t=N+m. For N+m+2≤t≤2N, one has

Cn,t;m[−N,N]=tt−n+m2−tt+n+m2+(N+1),−N≤n≤t−m−2(N+1),tt−n+m2,t−m−2N≤n≤2N−m−t,tt−n+m2−tt+n+m2−(N+1),2(N+1)−m−t≤n≤N.

For 2(N+1)≤t≤3N−m,

Cn,t;m[−N,N]=tt−n+m2−tt+n+m2+(N+1),−N≤n≤2N−m−t,tt−n+m2−tt+n+m2+(N+1)−tt+n+m2−(N+1),2(N+1)−m−t≤n≤t−m−2(N+1),tt−n+m2−tt+n+m2−(N+1),t−m−2N≤n≤N.

For instance, the example shown in panel (a) of Figure 4 corresponds to the case N=16, t=10, and m=8. Therefore, one has

pn,10;8=0,−16≤n≤−4,cos(nθ)cos(8θ)2cos(θ)101018−n2,−2≤n≤14,cos(16θ)cos(8θ)2cos(θ)10101−100,n=16,

with θ=π/34 and *n* even. Elementary calculus proves that

∑n=−1616pn,10;8=1cos(8θ)2cos(θ)10∑k=0810kcos(2(k−1)θ)+9cos(16θ)=1−1cos(8θ)2cos(θ)1010cos(16θ)+cos(18θ)−9cos(16θ)=1,

since cos(18θ)=−cos(16θ).
